# An Unusually Large Irritation Fibroma Associated with Gingiva of Lower Left Posterior Teeth Region

**DOI:** 10.1155/2016/5202181

**Published:** 2016-12-28

**Authors:** Ashish Lanjekar, Sunita Kulkarni, Sonali Akhade, Sonal Sonule, Usha Rathod

**Affiliations:** Department of Oral Medicine and Radiology, Swargiya Dadasaheb Kalmegh Smruti Dental College & Hospital, Nagpur, India

## Abstract

Fibroma is a benign tumor of oral cavity, with usually the tongue, gingiva, and buccal mucosa being the most common sites. Females are twice more likely to develop fibroma than males. The intraoral fibroma typically is well demarcated; and its size can vary from millimeter to few centimeters. Intraorally the growth is attached to the mucosa by means of a peduncle. Fibroma is generally slow growing, painless, smooth surface lesion and the color is slightly paler than the adjacent healthy tissue. Treatment usually requires total excision and recurrence is rare. Here we present a case of 37-year-old female patient reported to the Department of Oral Medicine and Radiology with the chief complaint of a growth in the lower left posterior teeth region 3 months earlier.

## 1. Introduction

Intraoral localized reactive lesions occurring on the gingiva are common which include irritation/traumatic fibroma, peripheral ossifying fibroma, focal fibrous hyperplasia, pyogenic granuloma, inflammatory hyperplasia, and peripheral giant cell granuloma [1]. Irritation fibroma is the most common response of submucosa secondary to trauma from teeth or dental prosthesis. First case of intraoral fibroma was reported in 1846 called fibrous polyp and polypus [2]. Fibroma of gingiva is clinically presented as slow growing, well-demarcated growth, usually with normal colored mucosa and smooth surface, sessile or pedunculated base, and hard consistency [3], causing difficulty in mastication and speech. The size of the growth is generally smaller than 1.5 cm [4], although there are few reports of 4–6 cm [5]. Irritation fibroma is most commonly prevalent in anterior region and usually associated with interdental papilla. Cases in the posterior teeth region are rare in the literature. This paper reports the case of irritation fibroma of 37-year-old female associated with gingiva of lower left posterior teeth region.

## 2. Case Report

### 2.1. Clinical Examination

A 37-year-old female patient was reported to the Department of Oral Medicine and Radiology with the chief complaint of a growth in the lower left posterior teeth region 3 months earlier. She was apparently alright 3 months back when she experienced food lodgment and pain in the area of chief complaint when she was using tooth pick to remove the lodged food particles. She noticed sometimes bleeding after using toothpick. After few days she noticed a small pea sized growth in the region. The growth progressively increased to present size with no history of pain bleeding and paresthesia. She gives exfoliation of one tooth during the period of enlargement of the growth. The medical history was not contributory.

**Figure 1:**
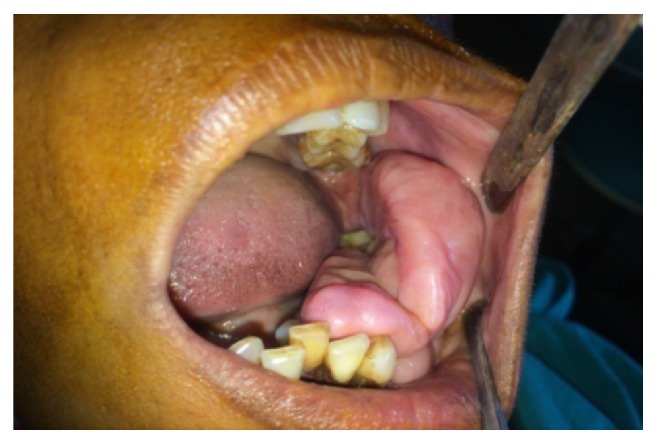
Intraoral preoperative lesion.

**Figure 2:**
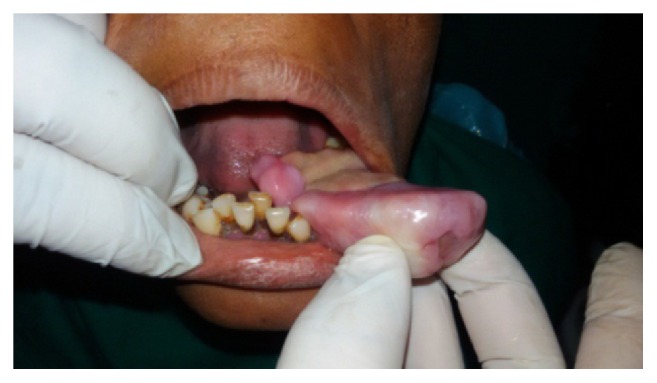
Intraoral preoperative lesion.

**Figure 3:**
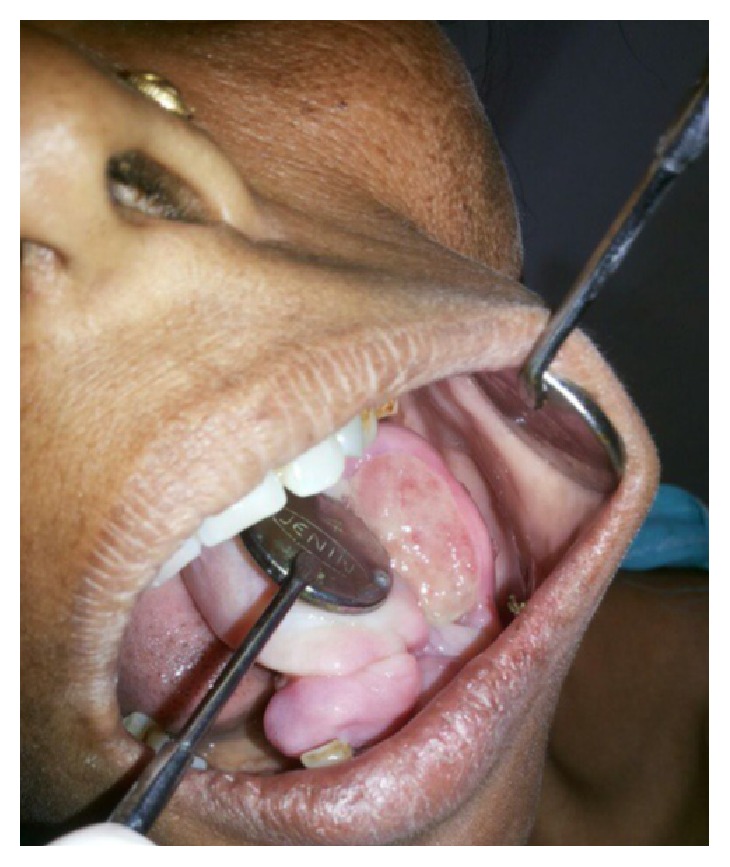
Intraoral preoperative lesion.

Intraoral clinical examination revealed a well-defined, exophytic, multilobulated growth over the mandibular alveolar ridge in region of lower left first molar, measuring approximately 5 cm × 4 cm × 3 cm in size, extending mesiodistally from mesial surface left lower canine up to the distal surface of lower left second molar. Surface of the lesion was smooth, shiny, and with normal color of oral mucosa (Figures 1 and 2). Small ulcerative lesion was seen on the buccal aspect of the growth measures approximately 1 cm × 2 cm in diameter (Figure 3). The growth was firm in consistency, with pedunculated base, nontender, and noncompressible on palpation, no bruit or pulse was felt, temperature was not raised, and it was movable over the peduncle. On the basis of history clinical presentation a provisional diagnosis of irritation fibroma was given.

### 2.2. Investigations

**Figure 4:**
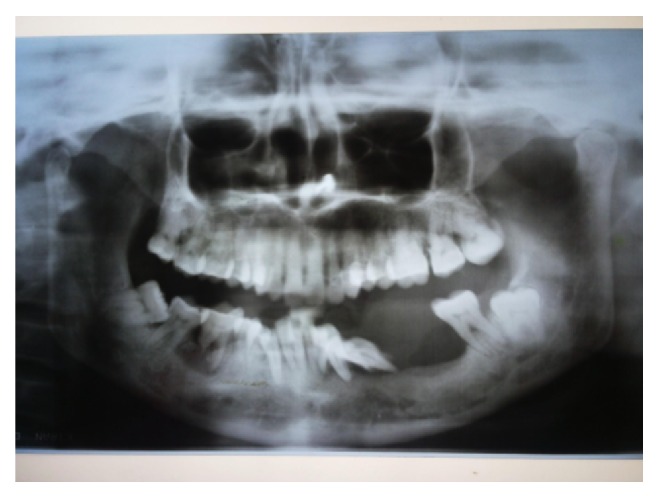
Orthopantomograph.

Orthopantomograph was obtained. The radiographic examination shows the missing left lower first molar, mesially displaced, and floating tooth appearance with lower left first and second premolars. There is generalized bone loss in interdental region suggestive of generalized moderate periodontitis. No radiographic abnormality was detected in the bone related to the region associated with the lesion (Figure 4).

**Figure 5:**
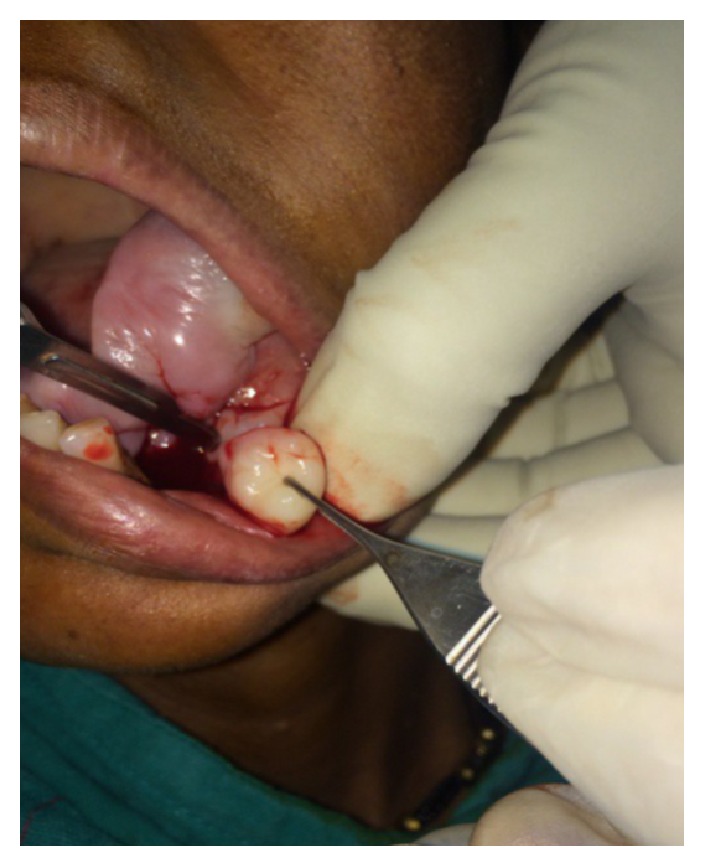
Incisional biopsy.

**Figure 6:**
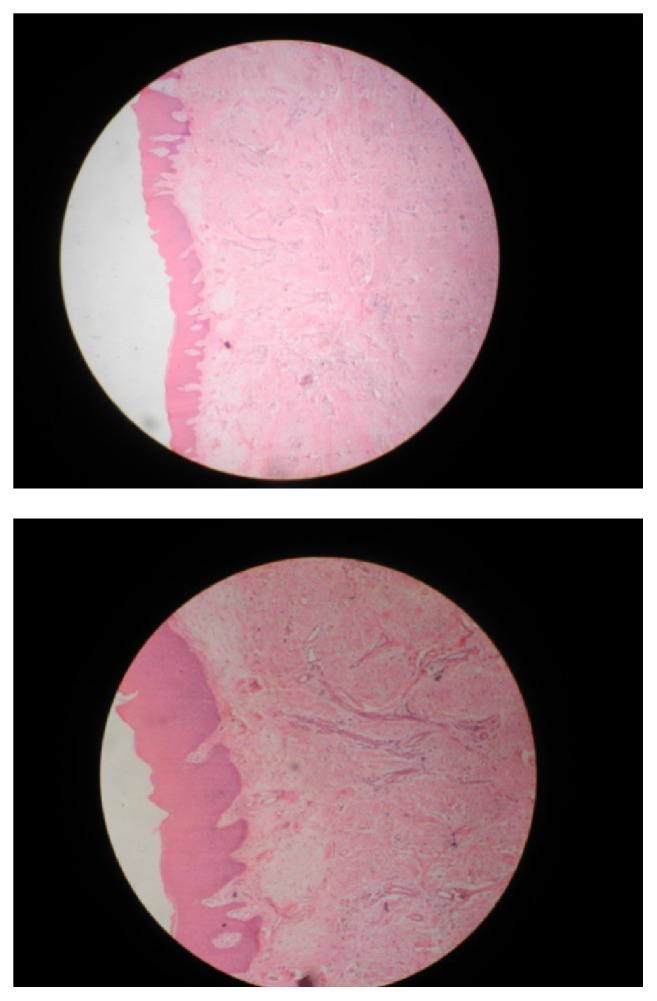
Histopathology.

Routine hematological examinations including hemogram, blood sugar level, HBsAg, and HIV screening tests were found to be within normal physiological limits. The histopathological report of incisional biopsy (Figure 5) shows connective tissue mass lined by parakeratinized stratified squamous epithelium of variable thickness. Some parts of tissue show myxoid areas with delicate fibers and stellate shaped cells; multinucleated giant cells are also visible (Figure 6).

### 2.3. Diagnosis

The differential diagnosis consisted of pyogenic granuloma, periapical giant cell granuloma, aggressive gingival fibromatoses, odontogenic myxoma, periapical odontogenic fibroma, and giant cell fibroma. These outcomes were discussed with the patient in an attempt to alleviate fear of carcinoma. Thus, a final diagnosis was considered as an irritation fibroma.

### 2.4. Treatment

**Figure 7:**
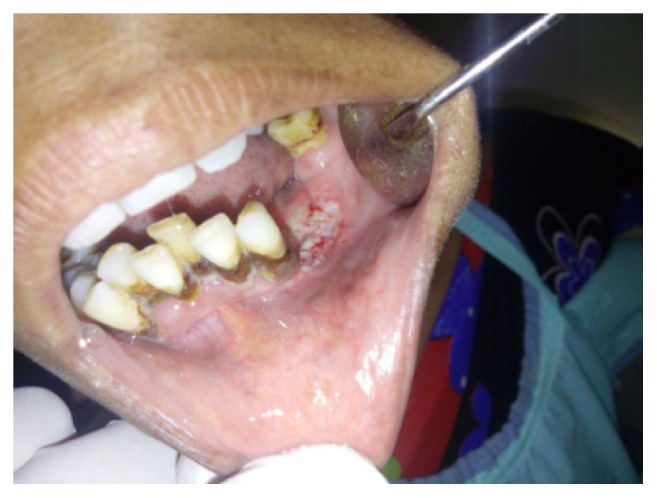
Eight-day postoperative photograph.

The lower first and second molars were extracted. Total surgical excision of the lesion under local anesthesia and aseptic conditions was done. It was followed up after 8 days showing healing at surgical site of excision (Figure 7).

## 3. Discussion

Inflammatory hyperplasia is different from irritation fibroma that histologically represents inflamed fibrous tissue along with granulation tissue [6, 7]. Reactive hyperplastic masses vary in size and depend upon the inflammatory components. Similar gingival lesions are often referred to as an epulis [6, 8]. Irritation fibroma develops more commonly in females than in males. It develops frequently between second and fourth decades of life. The high female predilection and a peak occurrence in the second decade of life suggested hormonal influences.

Approximately 60% of irritation fibromas occur in the maxilla and they are found more often in the anterior region, with 55–60% presenting in the incisor-cuspid region, rare in posterior region [9], but in our case the fibroma is present in posterior region of mandible. It is usually reported with the diameter of 1.5 rarely reaching 3 cm; very few case reports are present in the literature with the lesion measures about 6–9 cm [10]. The present case was of 5 cm × 4 cm × 3 cm in diameter with ulcerated surface.

Histopathologically, irritation fibroma can appear as an intact or ulcerated stratified squamous epithelium along with shortening and flattening of rete pegs. Treatment of irritation fibroma consists of elimination of etiological factors, scaling of adjacent teeth, and total aggressive surgical excision along with involved periodontal ligament and periosteum to minimize the possibility of recurrence. Any identifiable irritant such as an ill-fitting dental appliance and rough restoration should be removed [10]. Long-term postoperative follow-up is extremely important because of the high rate of reoccurrence of incompletely removed lesion.
